# Application of the Luminescent *lux*CDABE Gene for the Rapid Screening of Antibacterial Substances Targeting *Pseudomonas aeruginosa*

**DOI:** 10.3390/foods12020392

**Published:** 2023-01-13

**Authors:** Yue Peng, Qian Wang, Kaixiang Zhu, Wu Ding

**Affiliations:** 1 College of Food Science and Engineering, Northwest A&F University, Xianyang 712100, China; 2 Academy of National Food and Strategic Reserves Administration, Beijing 100037, China; 3 College of Life Sciences, Northwest A&F University, Xianyang 712100, China

**Keywords:** *lux*CDABE, *Pseudomonas aeruginosa*, bacteriostatic substances, rapid

## Abstract

*Pseudomonas aeruginosa* (*P. aeruginosa*) is a typical Gram-negative bacterium that can cause the spoilage of catered food products. Using a luminescent reporter gene (*lux*CDABE), this study sought to construct a cell-based biosensor (PAO1-CE) to rapidly screen antibacterial substances against *P. aeruginosa*. A total of six antibiotics belonging to five categories were used as the model test substances. The results of the bioluminescence detection method were verified using traditional antibacterial research assessments. The correlation coefficient of the regression equation fitting the data generated using this method was greater than 0.98, supporting the credibility of this approach. Additionally, the EC_50_ of each of the antibiotics assessed in this study was lower than the 1/2 MIC determined by conventional means. All six of the antibiotics caused varying degrees of damage to the cell membrane and cell wall of *P. aeruginosa*. Importantly, this novel method helped shorten the time necessary for active-compound detection and could be used for high-throughput detection, which would also help improve the detection efficiency. The application of this method towards the discovery of novel antibacterial compounds targeting *P. aeruginosa* holds substantial promise for greatly improving the efficiency of compound discovery.

## 1. Introduction

As spoilage bacteria, the Gram-negative *Pseudomonas aeruginosa* (*P. aeruginosa*) is a major contributor to the spoilage of aquatic products, meats, and cold ready-to-eat foods [1,2,3]. Simultaneously, *P. aeruginosa* is also a conditional pathogen and a common agent causing chronic infection in immunocompromised patients [4,5]. Moreover, these bacteria often cause respiratory, urinary tract, and intestinal infections, and provoke the formation of green pus in wounds [6,7,8,9]. Therefore, *P. aeruginosa* contamination has become an important factor in regard to human health [10,11]. However, a method to quickly screen and find substances that can inhibit the growth of *P. aeruginosa* is currently unavailable.

At present, the detection of *P. aeruginosa* antibacterial substances is generally carried out using traditional methods, such as minimal inhibit concentration (MIC) detection, which is used to determine if a particular substance can inhibit the growth of a given microorganism [12,13,14,15]. This would then be followed by assessing changes in the morphology (via transmission electron microscopy, scanning electron microscopy, laser confocal microscopy) [16,17,18,19,20], metabolism [21,22,23], and other characteristics at effective concentrations of the identified compound. However, it takes several days for each measurement of the MIC. As such, it is not possible to quickly and efficiently screen a large number of antibacterial substances via this approach, which has delayed the discovery of new antibacterial compounds.

Bioluminescence is one of the biosensor techniques that exploits the emitted light released from the enzymatic reaction [24]. The mechanism of bioluminescence by which bacteria produce light is the same for all bioluminescence species. The chemical reaction is listed as follows:

(1) $${\mathrm{FMNH}}_{2}+O_{2}+\mathrm{RCHO}\overset{\mathrm{luciferase}}{\to }\mathrm{FMN}+H_{2}O+\mathrm{RCOOH}+\mathrm{light}\;(490\;\mathrm{nm})$$

This light-emitting reaction is catalyzed by luciferase, a mixed-function oxidase, which catalyzes the oxidation of reduced flavin mononucleotides (FMNH_2_) and long-chain (typically 10–14 carbons) aliphatic aldehydes [25]. The core element that mediates this reaction is the *lux* gene cluster. Among the *lux* gene clusters, only five major genes (*lux*CDABE) are all present in every luminescent bacterium, and these define the core elements needed for bacteria to produce bioluminescence [26].

As a monitoring tool, the advantages of biosensors include their functional diversity, low cost, high recognition, high sensitivity, intellectualization, and portability. Thus, biosensors have become the focus of research in the field of rapid food safety detection. The *lux* gene is an efficient reporter gene, and its application in *P. aeruginosa* mainly focuses on the study of plankton and biofilm. Damron et al. [27] constructed a wide-host-range vector based on mini-Tn7 carrying the bioluminescence reporter *lux*CDABE. The construct is useful for the localization of bacteria during infections or for characterizing the regulation of promoters of interest in Gram-negative bacteria. Britta et al. [28] constructed a pBS110 vector using the *lux*CDABE gene cluster and obtained luminous *P. aeruginosa*. It proved to be a rapid, sensitive, and reproducible system for screening reagents to inhibit bacterial adhesion.

However, the current application of the *lux* reporter gene in *P. aeruginosa* is focused on qualitative research, and there is no relevant report on the evaluation of the toxic effect of substances on *P. aeruginosa*. Based on this, a mathematical quantitative model for the rapid screening of *P. aeruginosa* antibacterial susceptibility was constructed using recombinant *P. aeruginosa* PAO1-CE (PAO1-CE) as a biosensor in this study, which constitutively produced bioluminescence, to help improve the efficiency and accuracy of evaluating test substances. A total of six antibiotics belonging to five categories were used as the model test substances (Str: streptomycin sulfate, aminoglycosides antibiotics; Car: carbenicillin, β-lactam antibiotics; Amp: ampicillin, β-lactam antibiotics; Ter: oxytetracycline hydrochloride, tetracycline antibiotics; Tmp: trimethoprim, sulfonamides antibiotics; and Cip: ciprofloxacin, quinolones antibiotics). Additionally, traditional antibacterial research methods were used to verify the sensitivity of *P. aeruginosa* to antimicrobial substances, including the assessment of the MIC, morphological changes, cell membrane damage, ATP content, and cell wall permeability. The results indicate that the new biosensor, PAO1-CE, could be used for the rapid screening of antibacterial substances targeting *P. aeruginosa*. This method greatly shortened the detection time and improved the detection efficiency for such compounds. As such, this study demonstrates the feasibility of this method for application in the food industry and provides theoretical guidance for the usage amount of *P. aeruginosa* antibacterial substances.

## 2. Materials and Methods

### 2.1. Construction of the PAO1-CE Biosensor

**Figure 1:**
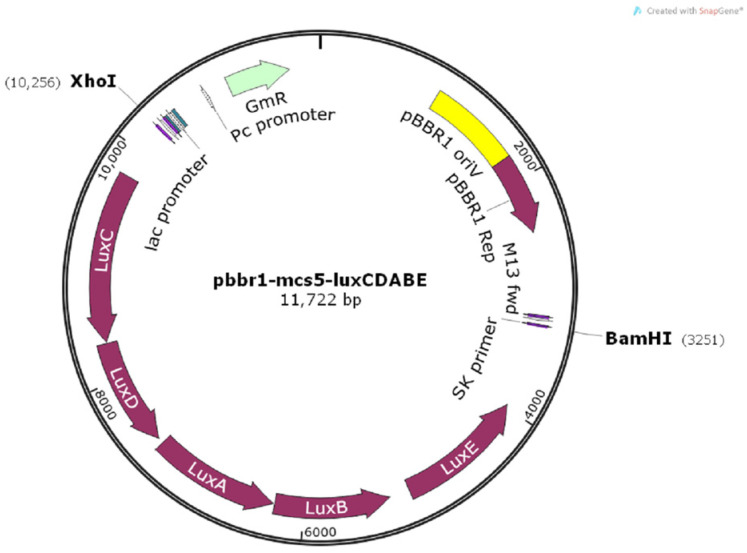
Construction of the pBBR1MCS-5-*lux*CDABE plasmid harboring the *lux*CDABE gene using *Bam*HI and *Xho*I.

PAO1-CE is a *P. aeruginosa* strain PAO1 containing the pBBR1MCS-5-*lux*CDABE plasmid (Figure 1). The whole sequence of the pBBR1MCS-5-*lux*CDABE plasmid is supplied in Supplementary Material S1. The gene cassette *lux*CDABE was obtained from the plasmid pBBR1MCS-4-*lux*CDABE. In this study, plasmid pBBR1MCS-5-*lux*CDABE was chosen because Amp could not screen out recombinant *P. aeruginosa*. The plasmids pBBR1MCS-5 (BioVector Science Lab Inc., Beijing, China) and pBBR1MCS-4-*lux*CDABE (BioVector Science Lab Inc., Beijing, China) were both cut with BamHI (G|GATTC) and XhoI (C|TCGAG; Thermo Scientific, Waltham, MA, USA), ligated using T4 DNA ligase (Thermo Scientific, USA), and transferred into *E. coli* TG1 competent cells. The plasmid pBBR1MCS-5-*lux*CDABE was then transferred into *P. aeruginosa* PAO1 (ATCC 15692, donated by Prof. Xihui Shen from College of Life Sciences, Northwest A&F University, China) using a Bio-rad electrotransformation unit (MicroPluser, Hercules, CA, USA) to obtain PAO1-CE. The detailed transformations procedures are supplied in Supplementary Material S2.

### 2.2. Chemicals

All antibiotics (purity > 99%) were provided by Beijing Solarbio Science & Technology Co. (Beijing, China). Tryptone, yeast extract, and agar power were supplied by Oxoid Inc. (Hants, UK). All other reagents were of analytical grade. Additionally, 25% glutaraldehyde, phosphate-buffered solution (PBS, 1×), and anhydrous alcohol (99%) were supplied by Aladdin (Shanghai, China). Double-deionized water (DDW; 18 mΩ·cm) was prepared using a Milli-Q system (Millipore Co., Boston, MA, USA).

### 2.3. Preparation of the Test Solutions Containing Antibioticss

Stock solutions of Amp, Str, and Car were prepared separately by dissolving 1 g of the standard in 10 mL of distilled water. Stock solutions of Tmp and Cip were prepared using 20% (*v*/*v*) acetic acid (HAc). A stock solution of Ter was prepared using 20% (*v*/*v*) HCl. All the stock solutions were passed through a 0.22 μm membrane filter and stored at 4 °C. The following range of concentrations of the test solutions were prepared by diluting with distilled water: Str (50.00 μg/mL, 25.00 μg/mL, 12.50 μg/mL, 6.75 μg/mL, and 1.00 μg/mL); Car (47.50 μg/mL, 23.75 μg/mL, 11.875μg/mL, 5.9375 μg/mL, and 1.00 μg/mL); Amp (1800.00 μg/mL, 900.00 μg/mL, 450.00 μg/mL, 225.00 μg/mL, 112.50 μg/mL, and 56.25 μg/mL); Ter (100.00 μg/mL, 50.00 μg/mL, 25.00 μg/mL, 12.50 μg/mL, and 6.75 μg/mL); Tmp (2000.00 μg/mL, 1600.00 μg/mL, 1200.00 μg/mL, 1000.00 μg/mL, 800.00 μg/mL, and 500.00 μg/mL); and Cip (0.70 μg/mL, 0.35 μg/mL, 0.175 μg/mL, 0.10 μg/mL, and 0.05 μg/mL).

### 2.4. Inhibition Ratio (IR) of the Six Antibiotics on Luminescence

A single colony of PAO1-CE was inoculated into 50 mL of Luria–Bertani (LB) liquid medium (containing 50 μg/mL kanamycin and 100 μg/mL gentamicin). The bacterial culture was grown overnight (37 °C, 180 rpm) to stationary phase (OD_600nm_ = 1.8). The bacterial culture was then transferred to fresh LB medium with an inoculum size of 1% (*v*/*v*) and incubated under the same conditions (37 °C, 180 rpm) for 7 h until the bioluminescence intensity of the bacterial culture reached the maximum value. The culture was centrifuged at 5000× *g* for 5 min and resuspended with fresh LB medium to OD_600nm_ = 1.0 before cytotoxicity testing. A total of 200 μL of the above culture medium containing the different antibiotic concentrations (with an equal volume of distilled water as the negative control) were added into each well of a black 96-well plate. The bioluminescence intensity was measured with a Victor X3 luminescence detector (PerkinElmer, Waltham, MA, USA) at 37 °C. Each treatment was carried out in triplicate, and the IR after a 2 h exposure was calculated as follows:

(2) $$\mathrm{IR}=\frac{L_{\mathrm{CK}}-L}{L_{\mathrm{CK}}}\times 100$$

where IR: inhibition ratio of luminescence (%); L_CK_: luminescence intensity of the negative controls (distilled water) (count per second (CPS)); and L: luminescence intensity of the samples (CPS).

### 2.5. Traditional Methods for Verifying the Sensitivity of Antibacterial Substances

#### 2.5.1. MIC

Tryptic soybean peptone agar (TSA) was aseptically transferred into sterile 24-well plates containing the different antibacterial substances. The contents (the final volume was 1 mL) of each well were gently mixed. The final concentrations of each antibacterial substance were between 4 mg/mL to 0.5 μg/mL. After being solidified, the agar in each well was spotted with 2 μL (approximately 10^4^ CFU) of the test bacterium, and the plates were then incubated at 37 °C for 24 h. The lowest concentration of an antibacterial substance that resulted in no visible growth of the test bacterium was considered the MIC.

#### 2.5.2. Growth Curve and Influence of the Dynamic Growth Model

The growth curves were constructed using the method of Shi et al. [20] with minor modifications. An overnight seed culture of PAO1-CE was centrifuged at 5000× *g* for 5 min to collect the cells. The OD_600nm_ was adjusted to 0.5 (approximately 1.5 × 10^8^ CFU/mL) with fresh LB medium. Each antibacterial substance was then dissolved with the bacterial solution above (OD_600nm_ = 0.5) and added to the cultures to obtain final concentrations of 0 MIC, 1/32 MIC, 1/16 MIC, 1/8 MIC, 1/4 MIC, 1/2 MIC, and 1 MIC. The 0 MIC was used as the positive control (CK group), and uninoculated LB served as the negative control. Then, 300 μL of the culture was transferred into each well on a honeycomb plate (Bioscreen C, Helsinki, Finland). Cell growth was monitored using an automatic growth curve analyzer (Bioscreen C; Oy Growth Curves Ab, Helsinki, Finland) at 37 °C every 1 h at 600 nm. All analyses were conducted in triplicate. With time (h) as the abscissa and the suspension OD_600nm_ as the ordinate, a growth curve was constructed. The modified Gompertz model was selected to fit the growth conditions and characterize the growth parameters of this strain. The modified Gompertz’s model can be expression as:

(3) $${\mathrm{OD}}_{t}=A+(B-A)\left\{-\exp\left[-\mu \left(t-M\right)\right]\right\}$$

(4) $$\lambda =M-(1/\mu ){\mathrm{OD}}_{t}=A+(B-A)\left\{-\exp\left[-\mu \left(t-M\right)\right]\right\}$$

(5) $$\mu_{\max}=\left(B-A\right)\mu /e$$

where OD_t_: the concentration of the bacterial liquid at *t*; *B*: the maximum bacterial concentration; *A*: the initial bacterial concentration; *M*: the time (h) used for the strain to reach the stationary phase; μ: relative growth rate during the exponential phase (ΔOD_600nm_/h); λ: lag phase (h); and μmax: the maximum growth rate (OD_600nm_/h).

The sigmoidal function available in the SigmaPlot software was used to fit the parameters of *A*, *B*, *M*, μ, λ, and *M*. The correlation coefficient R^2^ was used as the evaluation factor to gauge the fit of the model.

#### 2.5.3. Measurement of Intracellular ATP Concentrations

ATP is the most basic carrier for energy conversion in living systems, and its content is directly related to energy metabolism in most organisms. As a critical energy molecule, ATP plays a role in various physiological and pathological processes of the cell, and many functions of the cell are affected by changes in ATP levels [24]. Usually, when cells are apoptotic, necrotic, or in a toxic state, ATP levels decrease, while high glucose stimulation can increase intracellular ATP levels in some cells. The intracellular ATP concentration of PAO1-CE was analyzed according to the method described by Tian et al. [29], with some modifications. An overnight culture of PAO1-CE was harvested by centrifugation (5000× *g*, 5 min), and the cells were then resuspended in DDW to achieve an OD_600nm_ of 0.5 (approximately 4 × 10^8^ CFU/mL), with 2 mL of cell solution placed into Eppendorf tubes. Each antibiotic was then added to a tube at final concentrations of 0 MIC, 1 MIC, and 2 MIC, respectively. The samples were then incubated at 37 °C for 30 min. ATP was extracted via ultrasound (SCIENTZ-IID; Ningbo Scientz Biotechnology Co., Ningbo, China) on ice for 15 min. Then, the samples were centrifuged for 5 min at 5000× *g*, and the supernatant was taken and stored on ice to prevent ATP loss. ATP was measured using an ATP assay kit according to the manufacturer’s instructions (Nanjing Jiancheng Bioengineering Institute, Nanjing, China).

#### 2.5.4. Measurement of Cell Wall Permeability via AKP Activity

AKP is localized between the cell wall and cell membrane, and AKP activity cannot be detected in the culture medium under normal conditions [30]. As such, the content of AKP activity in the cells can be used as a measure of the cell wall permeability of a bacterium [21]. AKP assays were used to evaluate the effects of each antibiotic on the integrity of the cell wall. The alkaline phosphatase (AKP) concentration of PAO1-CE was analyzed using an AKP assay kit (Nanjing Jiancheng Bioengineering Institute, Nanjing, China). The pretreatment and active concentrations of the antibacterial substances used were the same as the measurement of ATP, except the ultrasonic step was omitted. The protein contents were determined using a total protein quantitative assay kit according to the manufacturer’s instructions (Nanjing Jiancheng Bioengineering Institute, Nanjing, China).

#### 2.5.5. Measurement of Cell Membrane Damage via Integrity

The bacterial cell membrane is a structural component of bacteria that serves as a physical barrier for the cell and also performs as an important structure with complex functions in the life of cells [31]. If the cell membrane is damaged, both small molecules and some macromolecules, such as DNA and RNA, can escape from the cell. Therefore, the extravasation of intracellular substances can be used as a good indicator for evaluating cell membrane integrity. Since DNA and RNA have strong absorptions at A_260nm_, the determination of this indicator can estimate the integrity of the cell membrane. Damage to the cell membrane was determined using the method described by Lin et al. [32] with some modifications. Pretreatment of the bacteria was the same as for the method of the growth curve mentioned above. The concentration levels of the antibacterial substances were 0 MIC, 1 MIC, and 2 MIC. Then, 200 μL of the sample was transferred into each well of a 96-well UV microtiter plate (Corning, NewYork, NY, USA). The absorbance at 260 nm was recorded via a multilabel plate reader (Victor X3; PerkinElmer, Waltham, MA, USA) at an interval of 1 h. All analyses were conducted in triplicate.

#### 2.5.6. Morphological Observation via FESEM

Observations were performed using a field emission scanning electron microscope (FESEM), as described, with some modifications [33]. The cells (OD_600nm_ = 0.5) were treated independently with each antibacterial substance at 0 MIC and 2 MIC. After incubation at 37 °C for 8 h, the cells were harvested by centrifugation (5000× *g*, 5 min) and washed twice with PBS (0.1 M, pH 7.0). Then, the cells were resuspended in 2.5% glutaraldehyde and kept at 4 °C for 24 h. After centrifugation, the cells were dehydrated in a water–alcohol gradient at various alcohol concentrations (30%, 50%, 70%, 80%, 90%, 100%, and 100%) for 10 min, respectively. Subsequently, 2 μL samples were dropped onto the smooth surface of a single throw wafer (5 mm × 5 mm × 0.1 mm; Harbin Tebo Technology Co., Harbin, China) and then dried using an automatic Critical Point Dryer (Leica EM CPD300; Leica Microsystems, Wetzlar, Germany) for 4 h. Finally, the wafers were fixed on a FESEM support and sputter-coated with gold under vacuum (Q105TS; Quorum Technologies Ltd., Laughton, UK); this was followed by microscopic examination using a FESEM (Nova Nano SEM-450; FEI Company, Hillsboro, OR, USA).

### 2.6. Statistical Analysis

All experiments were performed in triplicate. Statistical analyses were performed using SPSS software (version 20.0; SPSS, Inc., Chicago, IL, USA). The data were presented as the mean values ± standard error (SE, n = 3), and differences between means were tested using least significant difference (LSD) tests (*p* < 0.05, *p* < 0.01). The data were plotted using SigmaPlot 10.0 (Systat Software Inc., San Jose, CA, USA).

## 3. Results

### 3.1. Cytotoxicity of the Six Test Antibiotics Evaluated by PAO1-CE

**Figure 2:**
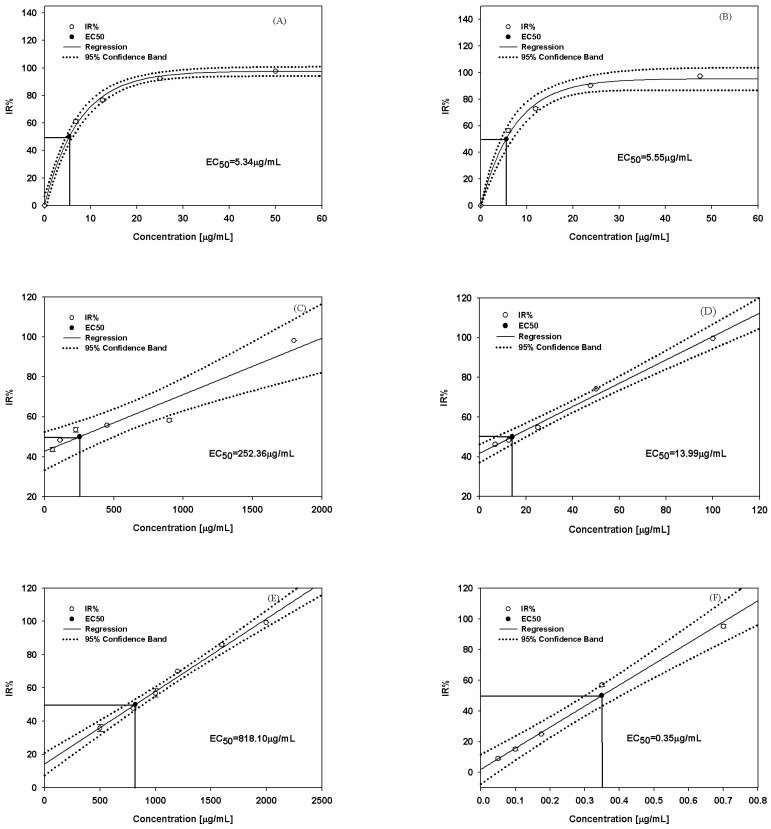
IR% of PAO1-CE at different concentrations of Str (**A**), Car (**B**), Amp (**C**), Ter (**D**), Tmp (**E**), and Cip (**F**). IR: luminescence inhibition ratio; EC_50_: concentration for 50% of maximal effect; Regression: fitted curve. Each point represents a mean value ± SE of three replicates.

A self-bioluminescence biosensor, PAO1-CE, was established using the host strain *P. aeruginosa* harboring the plasmid pBBR1MCS-5-*lux*CDABE. PAO1-CE was then applied to detect antibiotics targeting *P. aeruginosa*. A total of six antibiotics representing five different categories were used as the model test substances. The IR% values of PAO1-CE increased with the increasing concentrations of Str, Car, Amp, Ter, Tmp, and Cip, which exhibited a significant correlation with the concentrations presented in Figure 2 and Table 1. With these concentrations as the abscissa and the luminescence inhibition rate as the ordinate, a mathematical model was fitted to the data. As Figure 2 shows, the maximum concentration of Str/Car/Amp/Ter/Tmp/Cip used in this experiment is 50 μg/mL, 47.5 μg/mL, 1800 μg/mL, 100 μg/mL, 2000 μg/mL, and 0.7 μg/mL. At these concentrations, the IR% of PAO1-CE was 98.52%, 97.25%, 98.14%, 99.64%, 99.37%, and 95.04%, respectively. The sensitivity of the PAO1-CE biosensor was higher than that determined via MIC detection. The minimum concentration used in this experiment of Str (1 μg/mL), Car (1 μg/mL), Amp (56.25 μg/mL), Ter (6.75 μg/mL), Tmp (500 μg/mL), and Cip (0.1 μg/mL) also resulted in the inhibition of PAO1-CE bioluminescence (Figure 2). The R^2^ correlation coefficients of the mathematical models were both greater than 0.98, and detailed information regarding the six fitted curves is listed in Table 1. According to the EC_50_ (concentration for 50% of maximal effect) of the six measured antibiotics towards PAO1-CE, their cytotoxicity was in the order of Cip > Str > Car > Ter > Amp > Tmp.

### 3.2. Traditional Methods for Verifying the Sensitivity of Antibacterial Substances

#### 3.2.1. MIC

The six antibiotics showed different inhibitory effects against PAO1-CE. The MICs of Str, Car, Amp, Ter, Tmp, and Cip against PAO1-CE were 200.00 μg/mL, 190.00 μg/mL, 1800.00 μg/mL, 100.00 μg/mL, 2000.00 μg/mL, and 1.00 μg/mL respectively.

#### 3.2.2. Growth Curves and the Influence of the Dynamic Growth Model

**Figure 3:**
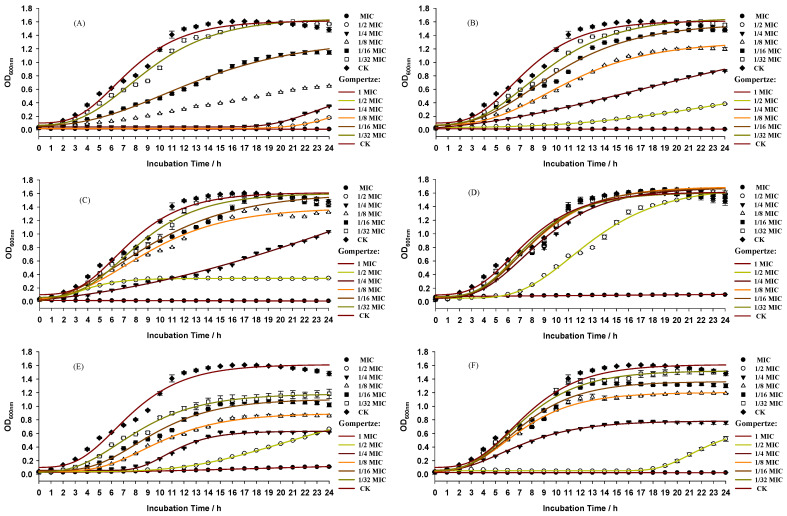
Growth curves for PAO1-CE cultured in LB with various concentrations of Str (**A**), Car (**B**), Amp (**C**), Ter (**D**), Tmp (**E**), or Cip (**F**).

When grown in media with one of the six antibiotics at MIC concentrations, the growth rate of *P. aeruginosa* was significantly decreased and completely inhibited (Figure 3). The modified Gompertz equation was applied to fit the growth curves and characterize the growth parameters, and the R^2^ of each fitting equation was greater than 0.98, indicating that this equation can accurately depict the growth of PAO1-CE. The growth parameters of the six antibiotics are listed in Table 2. Except for Ter, the μ_max_ of the other five antibacterial substances was significantly reduced (*p* < 0.05). The OD_max_ was also significantly reduced, except for Amp and Ter. The results of the μ_max_ and OD_max_ measurements indicate that concentration-dependent effects were clearly present.

#### 3.2.3. Intracellular ATP Concentrations

**Figure 4:**
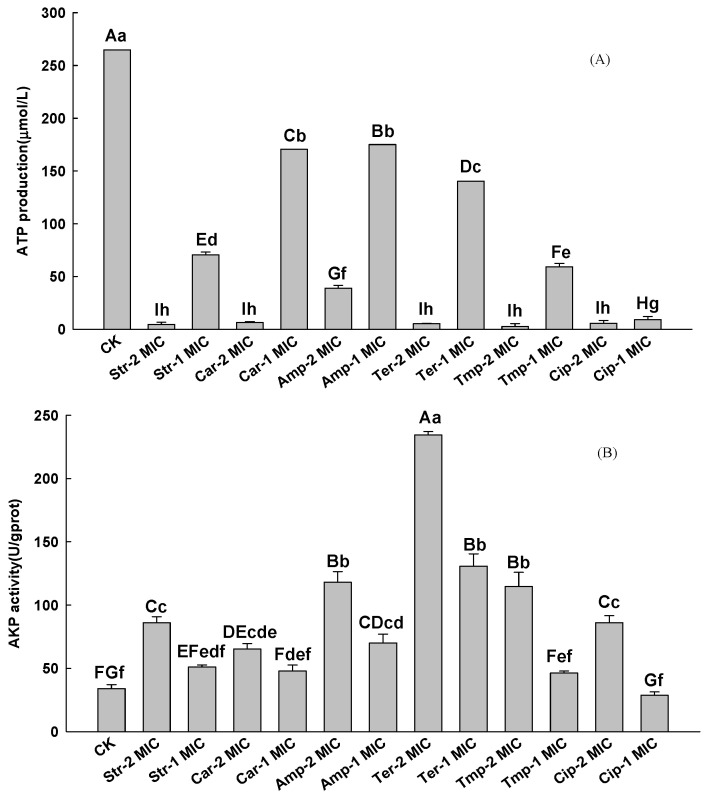
Effects of Str, Car, Amp, Ter, Tmp, and Cip on intracellular ATP production (**A**) and AKP activity (**B**) in PAO1-CE. The values represent the means ± SE (n = 3). Different letters indicate a significant difference between the groups (capital letter, *p* < 0.05; small letter, *p* < 0.01).

The effect of the six test antibiotics on the intracellular ATP concentrations is shown in Figure 4A. The intracellular ATP concentration in the CK group was 264.65 ± 0.10 μmol/L, while the Str/Car/Amp/Ter/Tmp/Cip at 1 MIC decreased to 70.44 ± 2.71 μmol/L, 170.34 ± 0.49 μmol/L, 174.96 ± 0.06 μmol/L, 140.31 ± 0.11 μmol/L, 59.26 ± 3.05 μmol/L, and 14.62 ± 1.99 μmol/L. Compared with the CK group, the six treatment groups showed a significant inhibition (*p* < 0.01) towards intracellular ATP concentrations in PAO1-CE. The influence of the six antibiotics on the intracellular ATP concentration of *P. aeruginosa* at 1 MIC from high to low is as follows: Cip > Tmp > Str > Ter > Car > Amp. As the concentration of action increased to 2 MIC, the intracellular ATP concentration of *P. aeruginosa* directly fell below 10% of the CK group and significantly decreased (*p* < 0.01).

#### 3.2.4. Cell Wall Permeability

The AKP activity results are presented in Figure 4B. The AKP activity of the CK group was 33.95 ± 3.09 U/gprot. At the MIC, the destructive effect of Str, Car, Tmp, and Cip on the cell wall was the same as that measured in the CK group (*p* > 0.05). However, the AKP activity of the Amp/Ter group increased to 70.14 ± 6.59 U/gprot and 130.71 ± 9.70 U/gprot, respectively, which was significantly higher than the CK group (*p* < 0.05). When the concentration increased to 2 MIC, the AKP activity of the Str/Car/Amp/Ter/Tmp/Cip group was significantly increased. The antibiotic that had the greatest influence on AKP activity was Ter, whose AKP activity increased 6.9 times.

#### 3.2.5. Cell Membrane Integrity

**Figure 5:**
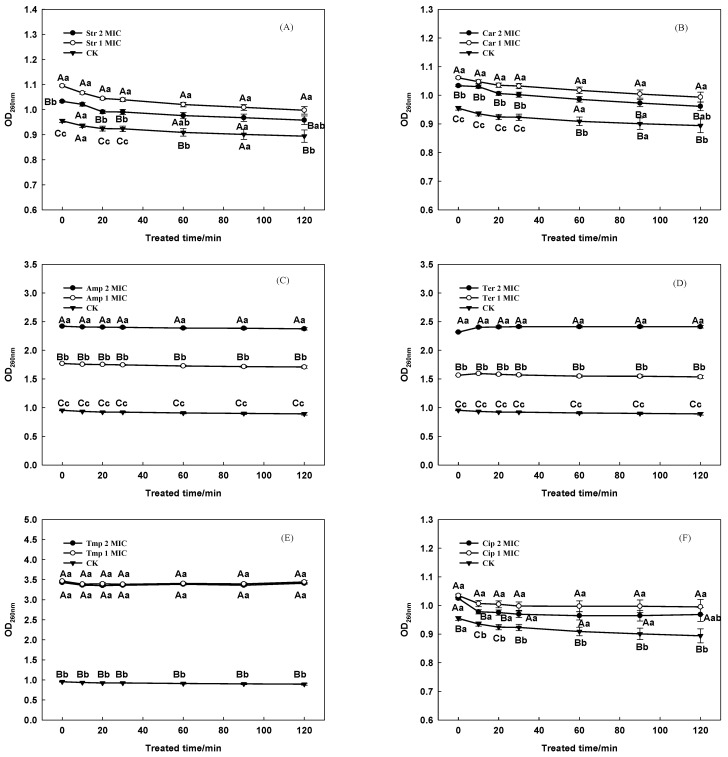
Effects of Str (**A**), Car (**B**), Amp (**C**), Ter (**D**), Tmp (**E**), and Cip (**F**) on cell membrane damage by PAO1-CE. Values represent the means ± SE (n = 3). Different letters mean a significant difference between the groups (capital letter, *p* < 0.05; small letter, *p* < 0.01).

The leakage of nucleotides from the bacterial cells treated with each antibiotic was significantly increased (*p* > 0.01) compared to the CK (Figure 5), indicating that these antibiotics could cause membrane permeabilization in PAO1-CE. As the treatment concentration increased from 1 MIC to 2 MIC, the leakage of nucleic acids in the Str (Figure 5A), Car (Figure 5B), Amp (Figure 5C), and Ter (Figure 5D) groups was significantly heightened, while the difference between 1 MIC and 2 MIC of the Tmp (Figure 5E) and Cip (Figure 5F) groups was not significant (*p* > 0.01). For Amp and Ter, as the concentration of action increased, the degree of destruction of the cell membrane of *P. aeruginosa* cells increased, and the effect of the treatment concentration of the two groups was significantly different (*p* < 0.01).

#### 3.2.6. Morphological Analysis via FESEM

**Figure 6:**
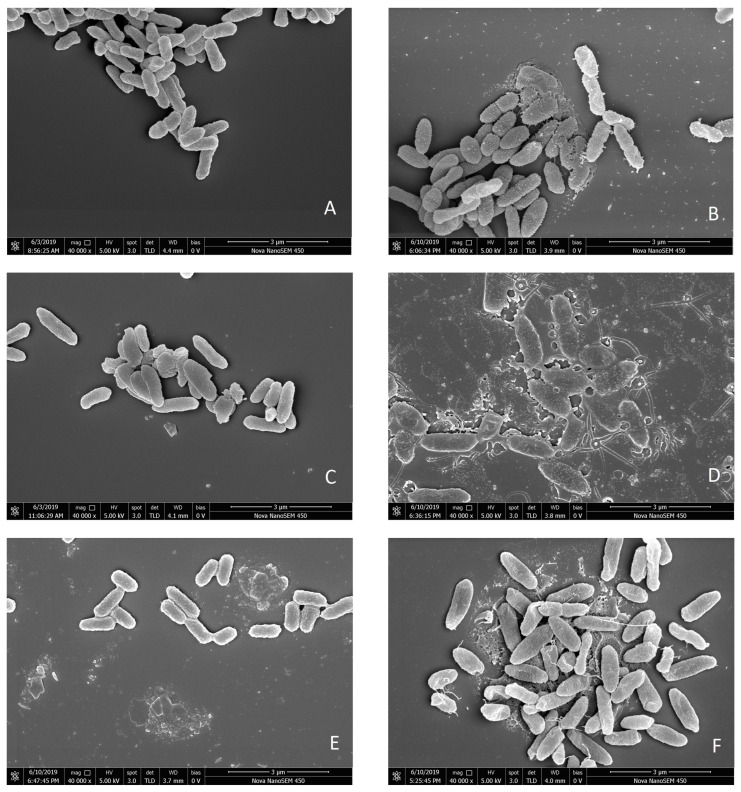

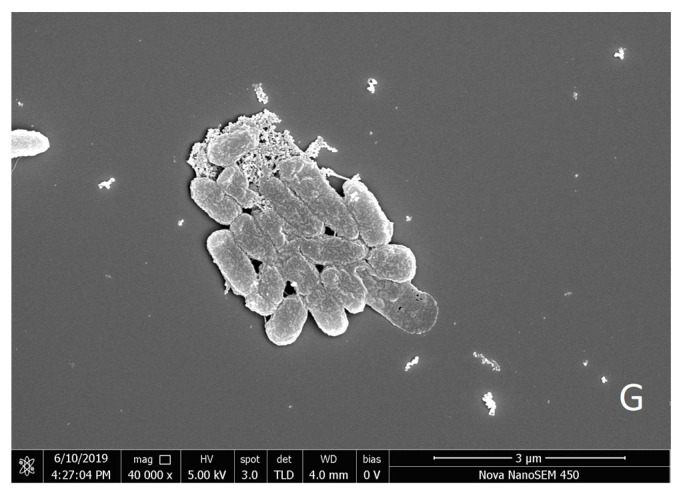
Scanning electron micrographs of untreated PAO1-CE (**A**) or PAO1-CE treated with Str (**B**), Car (**C**), Amp (**D**), Ter (**E**), Tmp (**F**), or Cip (**G**) at 2 MIC for 4 h.

Untreated cells are shown in Figure 6A. Comparing the control with treated cells (Figure 6B–G), the differences in morphology can be easily seen. The cells of the CK group showed a smooth surface and typical Gram-negative morphology (Figure 6A). In contrast, the cell surface of each treatment group presented with varying degrees of collapse. These phenomena demonstrate that treatment with Str, Car, Amp, Ter, Tmp, and Cip led to morphological changes in the cells. Comparing the degree of damage in each treatment group, Amp, Ter, and Tmp presented with more extensive visual damage to the cell compared to that of Str, Car, and Cip.

## 4. Discussion

### 4.1. Comparison of PAO1-CE with Other Whole-Cell Recombinant Bacterial Bioreporters

At present, the application of whole-cell biosensors in *P. aeruginosa* has been used for evaluating the mechanism of substance inhibition on *P. aeruginosa* growth, as well as contaminant detection in food, drugs, and cosmetics. These research studies adopted different plasmids or transposons to obtain their whole-cell biosensors of *P. aeruginosa*, including pKD-201/202/204/205/207 [34], pGLITE [35], pME4510-*lux* [36], pKD-*algU/pslM/pelA/algA/ppyR/bdlA* [37], pUTminiTn5*lux*CDABEKm2 [38], and pUC18-miniTn7TGm-*lux*CDABE [39]. The specific information regarding these studies is listed in Table 3. However, there is no effective mathematical model fitted to the biosensor data generated in the above studies. In this current study, we compared the relative changes in PAO1-CE luminescence inhibition under different concentrations of model antibiotics and obtained a strong positive correlation between the growth and luminescence expression of PAO1-CE (Table 1). These results help provide a solid foundation for the use of this biosensor in various *P. aeruginosa* research studies.

### 4.2. Cytotoxicity of the Six Test Antibiotics Verified by Traditional Methods

Based on the fact that PAO1-CE can evaluate the toxic effects of various model substances on *P. aeruginosa*, in order to clarify the ways in which various substances have toxic effects on them, the authors measured them using traditional microbial bacteriostatic research methods. 

The MIC values of Cip, Car, and Ter in this study were higher than those presented in previous studies (with MIC values of 0.06 μg/mL, >128 μg/mL, and 12 μg/mL, respectively) [40,41,42], which suggests the possibility that the resistance of *P. aeruginosa* towards these compounds has increased in recent years. According to the results of Figure 4A, six antibiotics had a significant effect on the intracellular ATP level of *P. aeruginosa*, while the expression of *lux*CDABE was closely related to the intracellular ATP level [43]. This proves that PAO1-CE could be used to evaluate the toxicity of compounds of *P. aeruginosa*. The AKP activity of the CK group and treatment groups was invariant between 1 MIC and 2 MIC, according to Figure 4B, which indicates an impaired function regarding cell wall permeability. However, as the antibiotic concentrations increased, the cell walls of all the treatment groups indicated significant damage compared with the CK group (*p* < 0.01). The results in Figure 5 show that the A_260nm_ of each group has almost no changes within 120 min after treatment with six antibiotics, indicating that the six antibiotics have damaged the integrity of the *P. aeruginosa* cell membranes, and this damage is caused by the action of the antibiotics themselves.

ATP is used for various cellular functions and is the basic carrier of energy conversion in all organisms. Its content is directly related to energy metabolism [44]. Usually, intracellular ATP levels decrease when cells are apoptotic, necrotic, or in a toxic state, and high glucose stimulation increases intracellular ATP levels in some cells. According to Figure 4A, six antibiotics significantly affected the intracellular ATP level of *P. aeruginosa*, and the expression of *lux*CDABE was inseparable from ATP [45]. This theoretically confirms that PAO1-CE could be used to evaluate the toxic effect of the test substance on *P. aeruginosa*.

The AKP enzyme is an enzyme that can dephosphorylate the corresponding substrate by hydrolyzing phosphomonoesters and producing PO_4_^3+^ and free OH^−^. Such substrate molecules include nucleic acids, proteins, alkaloids, etc. AKP is usually located between the cell wall and the cell membrane, and AKP enzyme activity is very low in the medium under normal conditions [32]. Therefore, AKP activity can be used as a measure of bacterial cell wall permeability [46]. According to the experimental results in Figure 4B, the presence of six antibiotics led to a rapid increase in AKP enzyme activity, indicating that all six antibiotics changed the cell wall permeability of *P. aeruginosa*.

The six antibiotics selected in this research belong to aminoglycosides (Str), β-lactams (Car; Amp), tetracyclines (Ter), sulfonamides (Tmp), and quinolones (Cip). Aminoglycoside antibiotics are glycoside antibiotics formed by the connection of amino sugars and aminocyclic alcohols through an oxygen bridge. Aminoglycoside, β-lactam, and tetracycline drugs all exert antibacterial effects by inhibiting the synthesis of bacterial proteins [47,48,49]. The protein synthesis in bacteria is related to many life activities and involves a large amount of ATP release. Therefore, Str, Car, Amp, and Ter have a significantly greater effect on the intracellular ATP level of *P. aeruginosa* than other antibiotics (Figure 4A). The principle of the antibacterial action of sulfonamides is to interfere with the folate metabolism of bacteria. It inhibits the activity of bacterial dihydrofolate reductase selectively, so that dihydrofolate cannot be reduced to tetrahydrofolate. As a coenzyme of one-carbon unit transferase, tetrahydrofolate is involved in the synthesis of nucleic acid precursors (purine, pyrimidine). The nucleic acid is an essential component for bacterial growth and reproduction [50]. However, the A_260nm_ of Tmp was higher than that of Cip, which means the amount of intracellular nucleic acid leakage of Tmp was higher than that of the Cip treatment group. The reason may be due to the inconsistency of the acute degree of inhibition of *P. aeruginosa* by Tmp and Cip. As the third-generation quinolone drugs, Cip has a nitrogen (hetero) bicyclic ring structure, which acts on bacterial cell DNA helicases, inhibits the synthesis and replication of bacterial DNA, and causes bacterial death [51]. This is consistent with the results of the A_260nm_ experiment that measures the integrity of the cell membrane in Figure 5. Cip destroys the integrity of the *P. aeruginosa* cell membrane, but its A_260nm_ value change is less than 0.2. This is because Cip directly inhibits the DNA synthesis of *P. aeruginosa*, making the treatment group’s nucleic acid leakage significantly lower than other antibiotics. 

According to the results utilizing the abovementioned antibiotics, it is clear that each of these compounds can cause damage to *P. aeruginosa* in regard to cell metabolism, cell membrane integrity, and cell wall permeability, suggesting that PAO1-CE could be used as a self-bioluminescence biosensor for rapidly detecting effective antibacterial substances targeting *P. aeruginosa*.

### 4.3. Analysis of Cytotoxicity Evaluation Mathematical Model Using PAO1-CE

At present, mathematical models are widely used in scientific research [32,52,53,54]. For different research samples, choosing an appropriate model to analyze and obtain a highly reliable research model can provide a certain degree of theoretical guidance for practical production applications. For the bioluminescence method to evaluate the toxicity of substances, the current research is placed more in environmental science, using various wild luminescent bacteria or recombinant luminescent bacteria to evaluate the toxicity of toxic and harmful substances in water or soil. Researchers used various wild luminescent bacteria or recombinant luminescent bacteria to evaluate the toxicity of toxic and harmful substances in water or soil [55,56]. Bello-López et al. [38] used the pUT miniTn5*lux*CDABEKm2 transposon to construct three recombinant luminescent bacteria to investigate the pollution of bagged platelet concentrates. The results showed that, in the logarithmic growth period, the linear correlation between the luminous intensity of the three bacteria and bacterial biomass R^2^ was 0.985, 0.976, and 0.981, respectively. This indicated that using the *lux*CDABE system to quantify luminescent activity at this stage is a fast and sensitive alternative method to study the propagation and automatic sterilization of bacterial contaminants in platelet concentrates. Shah and Naseby [36] determined the antiseptic efficacy of BKC by recombinant luminescent bacteria *P. aeruginosa* ATCC9027 tatH5-pME*lux*. The obtained correlation coefficient of the bioluminescence evaluation method was higher than 0.9, which was not significantly different from the results of the colony-forming units (CFUs) count. It also proved that lux+-tagged *P. aeruginosa* is the best construct for testing various antimicrobial agents.

The fitting model of Str and Car obtained in this study looks similar to the Gompertz model, but according to the comparison in Table 4, it is found that the decision coefficient of the Gompertz model is less than the exponential model: *y* = *a*(1 − exp(−*bx*)), which shows that the exponential model is more suitable for Str and Car samples. In the fitted exponential model, parameter a represents the limit IR% of the substance to *P. aeruginosa*, and b represents the relative growth rate of IR%. By comparing the parameter changes of Str and Car, it is concluded that, within the corresponding linear range, PAO1-CE is slightly more sensitive to Str dose changes than Car. 

The fitting models of Amp, Ter, Tmp, and Cip are linear models: *y* = *b* + *ax*. Parameter a is the relative growth rate of IR%, which represents the speed of the change in IR% with the acting dose; parameter b is a constant, and the value is the IR% of the PAO1-CE of the same volume of control when no dose of drug is added. 

According to the values of parameter a of each fitting model formula in Table 1, it could be determined that the sensitivity of PAO1-CE to Amp, Ter, Tmp, and Cip dose changes from high to low was Cip > Amp > Ter > Tmp within the corresponding linear range. Compared with the studies of Bello-López et al. [38] and Visaggio et al. [57], the detection method established in this study has a higher correlation. This also proves that PAO1-CE can be used to quantitatively evaluates the sensitive inhibition of *P. aeruginosa*. Recombinant luminescent PAO1-CE can be used to quickly evaluate whether *P. aeruginosa* has a toxic response to chemical substances. At the same time, it can also be used to study the distribution of *P. aeruginosa* in food systems, soil, or water bodies. It can intuitively obtain the migration and distribution of bacteria in infected objects without any damage in the whole process, which is helpful for developing effective control methods. For example, changes in luminous intensity play a role in the rapid evaluation of *P. aeruginosa* biofilm formation and fungicide efficiency.

## 5. Conclusions

In this study, the *lux*CDABE gene was used to construct the fluorescent biosensor PAO1-CE to rapidly screen six antibiotics that inhibit *P. aeruginosa*. The results of the bioluminescence detection method show that their cytotoxicity was in the order of Cip > Str > Car > Ter > Amp > Tmp. The six antibiotics all damaged the membrane and cell wall of *P. aeruginosa* to varying degrees, causing the extravasation of intracellular substances. AKP activity increased significantly, while intracellular ATP content decreased significantly. The FESEM results confirmed that all six antibiotics significantly changed the morphological appearance of *P. aeruginosa*. In addition, the EC_50_ of each antibiotic evaluated in this study was lower than the 1/2 MIC measured by conventional methods. The mathematical models fitted in this study also provide a reference for the subsequent discovery of other classes of bacteriostatic substances. As such, the luminescent bacteria test method using PAO1-CE appears to be promising for the evaluation of antibacterial effects targeting *P. aeruginosa* and the discovery of new bacteriostatic substances.

## Figures and Tables

**Table 1:** Comparative results between the PAO1-CE biosensor and MIC detection.

Antibiotics	Fitting Formula	R^2^	Liner Range (μg/mL)	EC_50_ (μg/mL)	1/2 MIC (μg/mL)
Str	y = 96.1138(1 − exp(−0.1393x))	0.9974	0.00~50.00	5.34	100.00
Car	y = 95.2735(1 − exp(−0.1362x))	0.9947	0.00~47.50	5.55	95.00
Amp	y = 42.8581 + 0.0283x	0.9243	56.25~1800.00	252.36	900.00
Ter	y = 41.7624 + 0.5885x	0.9932	6.75~100.00	13.99	50.00
Tmp	y = 14.311 + 0.436x	0.9925	500.00~2000.00	818.10	1000.00
Cip	y = 2.0228 + 137.28x	0.9889	0.05~0.7	0.35	0.50

**Table 2:** Kinetic parameters of PAO1-CE during growth in LB with different concentration of Str, Car, Amp, Ter, Tmp, or Cip.

Antibiotics	Concentrations	Growth Parameters		
		λ ± SE	μ_max_ ± SE	OD_max_ ± SE
Str	1/2 MIC	23.106 ± 0.251 ^A^	0.097 ± 0.001 ^C^	0.178 ± 0.011 ^E^
	1/4 MIC	19.432 ± 0.452 ^B^	0.069 ± 0.008 ^D^	0.352 ± 0.008 ^D^
	1/8 MIC	2.720 ± 0.154 ^D^	0.033 ± 0.001 ^E^	0.647 ± 0.013 ^C^
	1/16 MIC	3.874 ± 0.151 ^C^	0.074 ± 0.002 ^D^	1.155 ± 0.027 ^B^
	1/32 MIC	3.361 ± 0.233 ^CD^	0.146 ± 0.002 ^B^	1.603 ± 0.001 ^A^
	CK	3.124 ± 0.036 ^CD^	0.177 ± 0.001 ^A^	1.610 ± 0.005 ^A^
Car	1/2 MIC	12.071 ± 0.341 ^A^	0.030 ± 0.001 ^F^	0.384 ± 0.004 ^E^
	1/4 MIC	2.724 ± 0.498 ^C^	0.045 ± 0.001 ^E^	0.875 ± 0.011 ^D^
	1/8 MIC	4.182 ± 0.268 ^B^	0.095 ± 0.002 ^D^	1.211 ± 0.020 ^C^
	1/16 MIC	3.347 ± 0.017 ^BC^	0.120 ± 0.000 ^C^	1.485 ± 0.008 ^B^
	1/32 MIC	3.387 ± 0.104 ^BC^	0.144 ± 0.002 ^B^	1.607 ± 0.008 ^A^
	CK	3.124 ± 0.036 ^C^	0.177 ± 0.001 ^A^	1.610 ± 0.005 ^A^
Amp	1/2 MIC	1.335 ± 0.030 ^D^	0.061 ± 0.001 ^D^	0.349 ± 0.004 ^D^
	1/4 MIC	6.835 ± 0.462 ^A^	0.066 ± 0.002 ^D^	1.035 ± 0.011 ^C^
	1/8 MIC	2.406 ± 0.026 ^C^	0.108 ± 0.006 ^C^	1.483 ± 0.061 ^B^
	1/16 MIC	1.433 ± 0.173 ^D^	0.114 ± 0.002 ^C^	1.563 ± 0.015 ^AB^
	1/32 MIC	3.265 ± 0.097 ^B^	0.161 ± 0.009 ^B^	1.593 ± 0.002 ^A^
	CK	3.124 ± 0.036 ^B^	0.177 ± 0.001 ^A^	1.610 ± 0.005 ^A^
Ter	1/2 MIC	6.973 ± 0.024 ^A^	0.142 ± 0.004 ^E^	1.585 ± 0.014 ^B^
	1/4 MIC	3.590 ± 0.035 ^BC^	0.164 ± 0.001 ^D^	1.646 ± 0.002 ^A^
	1/8 MIC	3.462 ± 0.055 ^C^	0.175 ± 0.001 ^C^	1.653 ± 0.005 ^A^
	1/16 MIC	3.627 ± 0.016 ^B^	0.182 ± 0.002 ^AB^	1.655 ± 0.010 ^A^
	1/32 MIC	3.498 ± 0.086 ^BC^	0.185 ± 0.002 ^A^	1.611 ± 0.033 ^AB^
	CK	3.124 ± 0.036 ^D^	0.177 ± 0.001 ^BC^	1.610 ± 0.005 ^AB^
Tmp	1/2 MIC	12.237 ± 0.145 ^A^	0.053 ± 0.000 ^F^	0.665 ± 0.002 ^E^
	1/4 MIC	8.136 ± 0.024 ^B^	0.096 ± 0.001 ^D^	0.623 ± 0.003 ^E^
	1/8 MIC	4.956 ± 0.008 ^C^	0.086 ± 0.000 ^E^	0.869 ± 0.016 ^D^
	1/16 MIC	4.377 ± 0.023 ^D^	0.108 ± 0.001 ^C^	1.077 ± 0.022 ^C^
	1/32 MIC	2.870 ± 0.269 ^E^	0.116 ± 0.005 ^B^	1.203 ± 0.064 ^B^
	CK	3.124 ± 0.036 ^E^	0.177 ± 0.001 ^A^	1.610 ± 0.005 ^A^
Cip	1/2 MIC	18.456 ± 0.230 ^A^	0.091 ± 0.003 ^D^	0.521 ± 0.035 ^E^
	1/4 MIC	1.922 ± 0.162 ^D^	0.079 ± 0.002 ^E^	0.786 ± 0.013 ^D^
	1/8 MIC	2.524 ± 0.251 ^C^	0.134 ± 0.006 ^C^	1.200 ± 0.009 ^C^
	1/16 MIC	2.832 ± 0.098 ^BC^	0.151 ± 0.004 ^B^	1.365 ± 0.006 ^B^
	1/32 MIC	2.825 ± 0.095 ^BC^	0.167 ± 0.002 ^A^	1.541 ± 0.040 ^A^
	CK	3.124 ± 0.036 ^B^	0.177 ± 0.001 ^A^	1.610 ± 0.005 ^A^

Values with a different letter for the same antibiotic indicate a significant difference based on CK group (LSD; *p* < 0.05).

**Table 3:** Summary of lux-tagged *P. aeruginosa* that have been presented in recent studies.

Plasmids/Transposon	Relevant Information	Application Areas	Reference
pKD-201/202/204/205/207	pMS402 containing *lasI/rhlI/lasR/rhlR/aprA/rhlA* promoter, pMS402 expression reporter plasmid carrying the promoterless *luxCDABE* gene	Evaluating environmental regulation of *P. aeruginosa* PAO1 Las and Rhl quorum-sensing systems.	[34]
pGLITE	pLITE27 containing the *lux*CDABE operon of *Xenorhabdus luminescens*	Susceptibility to antimicrobials.	[35]
pME4510-*lux*	pME4510 containing a lux cassette from pSB417	Correlation between bioluminescence properties and cell growth.	[36]
pKD-*algU/pslM/pelA/algA/ppyR/bdlA*	pMS402 containing *algU/pslM/pelA/algA/ppyR/bdlA* promoter	Screening a library of 36 herb extracts for inhibitory properties against these genes.	[37]
pUTmini*Tn5lux*CDABEKm2	mini*Tn5* promotor probe carrying the *lux* operon from *Photorhabdus luminiscens*	Studying the propagation and auto*-*sterilization of bacterial contaminants in platelet concentrates.	[38]
pUC18-miniTn7TGm-*lux*CDABE	Gm^r^ on mini-Tn7T; *lux*CDABE transcriptional fusion vector	Selected fungi producing antimicrobials.	[39]

**Table 4:** Gompertz model and exponential model fitted Str and Car bacteriostasis results.

Antibiotics	Model	Formula	Parameter			R^2^
			*a*	*k*	*b*	
Str	Gompertz	*y* = *a*e(−*b*exp(−*kx*))	95.9183	2.9382	0.2500	0.9819
	Exponential	*y* = *a*(1 − exp(−*bx*))	96.1138	-	0.1393	0.9974
Car	Gompertz	*y* = *a*e(−*b*exp(−*kx*))	91.7190	2.8206	0.2634	0.9733
	Exponential	*y* = *a*(1 − exp(−*bx*))	95.2735	-	0.1362	0.9947

## Data Availability

All data generated or analyzed during this study are included in this published article (and its supplementary information files).

## Supplementary Materials

The following supporting information can be downloaded at: https://www.mdpi.com/article/10.3390/foods12020392/s1, Supplementary Material S1: The whole sequence of the pBBR1MCS-5-*lux*CDABE plasmid; Supplementary Material S2: Detailed transformations procedures.
