# Supplementary material for: Application of the Luminescent luxCDABE Gene for the Rapid Screening of Antibacterial Substances Targeting Pseudomonas aeruginosa
Source: Foods. 2023 Jan 13;12(2):392. doi: 10.3390/foods12020392 (PMC9857705; doi:10.3390/foods12020392)
Supplement: Supplementary file 1 [file foods-12-00392-s001.zip › foods-2119381-supplementary/foods-2119381 - Supplementary material S1-done.pdf]

Supplementary Materials 1

Application of the Luminescent *lux*CDABE Gene for the Rapid Screening of Antibacterial Substances Targeting *Pseudomonas aeruginosa*

Yue Peng [1,†], Qian Wang [1,2,†], Kaixiang Zhu [3] and Wu Ding [1,*]

1 College of Food Science and Engineering, Northwest A&F University, Yangling 712100, China
2 Academy of National Food and Strategic Reserves Administration, Beijing 100037, China
3 College of Life Sciences, Northwest A&F University, Yangling 712100, China
* Correspondence: ding_wu@nwsuaf.edu.cn; Tel.: +86-15002926160
† These authors contributed equally to this work.

The whole sequence of the pBBR1MCS-5-*lux*CDABE plasmid

ctcgggccgtctcttgggcttgatcggccttcttgcgcatctcacgcgctcctgcggcggcctgtagggcaggctcatacccctgccgaaccgcttttgtcagccggtcggccac
ggcttccggcgtctcaacgcgctttgagattcccagcttttcggccaatccctgcggtgcataggcgcgtggctcgaccgcttgcgggctgatggtgacgtggcccactggtg
gccgctccagggcctcgtagaacgcctgaatgcgcgtgtgacgtgccttgctgccctcgatgccccgttgcagccctagatcggccacagcggccgcaaacgtggtctggtc
gcgggtcatctgcgctttgttgccgatgaactccttggccgacagcctgccgtcctgcgtcagcggcaccacgaacgcggtcatgtgcgggctggtttcgtcacggtggatgc
tggccgtcacgatgcgatccgccccgtacttgtccgccagccacttgtgcgccttctcgaagaacgccgcctgctgttcttggctggccgacttccaccattccgggctggccgt
catgacgtactcgaccgccaacacagcgtccttgcgccgcttctctggcagcaactcgcgcagtcggcccatcgcttcatcggtgctgctggccgcccagtgctcgttctctggc
gtcctgctggcgtcagcgttgggcgtctcgcgctcgcggtaggcgtgcttgagactggccgccacgttgcccattttcgccagcttcttgcatcgcatgatcgcgtatgccgcc
atgcctgcccctcccttttggtgtccaaccggctcgacgggggcagcgcaaggcggtgcctccggcgggccactcaatgcttgagtatactcactagactttgcttcgcaaagt
cgtgaccgcctacggcggctgcggcgccctacgggcttgctctccgggcttcgccctgcgcggtcgctgcgctcccttgccagcccgtggatatgtggacgatggccgcgag
cggccaccggctggctcgcttcgctcggcccgtggacaaccctgctggacaagctgatggacaggctgcgcctgcccacgagcttgaccacagggattgcccaccggctac
ccagccttcgaccacatacccaccggctccaactgcgcggcctgcggccttgccccatcaattttttaattttctctggggaaaagcctccggcctgcggcctgcgcgcttcgct
tgccggttggacaccaagtggaaggcgggtcaaggctcgcgcagcgaccgcgcagcggcttggccttgacgcgcctggaacgacccaagcctatgcgagtgggggcag
tcgaaggcgaagcccgcccgcctgccccccgagcctcacggcggcgagtgcgggggttccaagggggcagcgccaccttgggcaaggccgaaggccgcgcagtcgatc
aacaagccccggaggggccactttttgccggagggggagccgcgccgaaggcgtgggggaaccccgcaggggtgcccttctttgggcaccaaagaactagatataggg
cgaaatgcgaaagacttaaaaatcaacaacttaaaaaagggggggtacgcaacagctcattgcggcacccccgcaatagctcattgcgtaggttaaagaaaatctgtaattg
actgccacttttacgcaacgcataattgttgtcgcgctgccgaaaagttgcagctgattgcgcatggtgccgcaaccgtgcggcaccctaccgcatggagataagcatggcca
cgcagtccagagaaatcggcattcaagccaagaacaagcccggtcactgggtgcaaacggaacgcaaagcgcatgaggcgtgggccgggcttattgcgaggaaaccca
cggcggcaatgctgctgcatcacctcgtggcgcagatgggccaccagaacgccgtggtggtcagccagaagacactttccaagctcatcggacgttctttgcggacggtcca
atacgcagtcaaggacttggtggccgagcgctggatctccgtcgtgaagctcaacggccccggcaccgtgtcggcctacgtggtcaatgaccgcgtggcgtggggccagc
cccgcgaccagttgcgcctgtcggtgttcagtgccgccgtggtggttgatcacgacgaccaggacgaatcgctgttggggcatggcgacctgcgccgcatcccgaccctgta
tccgggcgagcagcaactaccgaccggccccggcgaggagccgcccagccagcccggcattccgggcatggaaccagacctgccagccttgaccgaaacggaggaatg
ggaacggcgcgggcagcagcgcctgccgatgcccgatgagccgtgttttctggacgatggcgagccgttggagccgccgacacgggtcacgctgccgcgccggtagcac
ttgggttgcgcagcaacccgtaagtgcgctgttccagactatcggctgtagccgcctcgccgccctataccttgtctgcctccccgcgttgcgtcgcggtgcatggagccgggc
cacctcgacctgaatggaagccggcggcacctcgctaacggattcaccgtttttatcaggctctgggaggcagaataaatgatcatatcgtcaattattacctccacggggag
agcctgagcaaactggcctcaggcatttgagaagcacacggtcacactgcttccggtagtcaataaaccggtaaaccagcaatagacataagcggctatttaacgaccctgc
cctgaaccgacgaccgggtcgaatttgctttcgaatttctgccattcatccgcttattatcacttattcaggcgtagcaccaggcgtttaagggcaccaataactgccttaaaaaa
attacgccccgccctgccactcatcgcagtcggcctattggttaaaaaatgagctgatttaacaaaaatttaacgcgaattttaacaaaatattaacgcttacaatttccattcgcc
attcaggctgcgcaactgttgggaagggcgatcggtgcgggcctcttcgctattacgccagctggcgaaagggggatgtgctgcaaggcgattaagttgggtaacgccag
ggttttcccagtcacgacgttgtaaaacgacggccagtgagcgcgcgtaatacgactcactatagggcgaattggagctccaccgcggtggcggccgctctagaactagtg

gatcccccgggctgcaggaattcctttaatccctttaattcctggattttttctccgaaatctgggagcgtataaacatctggactcgtataaatcagttgactggtttctatgccttc
tttgccacatattatatgtatatcaactaataccgtattattatcataacctatagacatagttccattgtattttagcgcgttctcaaattcttcttttgatggatacgatgatccatctc
cgtactttttcgttaacgatttgcttactggcttcacaagtctgccaatattaatctctaatcttttaaatttcccatttaaaaaaaatgccattgcttcacgctttttgttagcaaacgaa
aagtcagaacatgaataaagtgatacttcttctattcgagaatctccctcaaatttttcaaaatcacataacctcttcgaaagaaactcttcttctgtcatgccaaactttgcatcttta
tacccatcaactgccattgcaccaaagctaatcattcccaatgcaagcataactgccaatttttttcatgttatctccttttgctttgagccaataataacaatgagaacaatgttaag
ccaaccccataaacctctatcaagtacccaaatgtggatgttttggagaattttgtaaagtgtagatgtcattcagatttatataacgcaagcattccacttacaattaggcaaag
gatatcaactatcaaacgcttcggttaagcttaaagcacaccctttctgcgtcctcgtattgacgcgacgtaaaatttcaacgagcacgccgggatacttaccatattctctgctaa
ttatcccgacatcatcggtaacaataaatgctggataactggttgctgacgcatccatataactcatcaaccccggcgttccatcaggtacaggtttcaacgtttcaggatcaagc
gctcgcgcatatacccacggcggaacatgtttacgctgcatttcatcctcaaagaaacaagtgttgagttcaacttgattaaatatatctcggatctgactaatatcactgagatt
gaaagtatcaaataaaagatgattgaaatcatcacgtttcagagattctttttcgtaacttttccagccgcctccggttatgatataaaggcttttatctccagaaaatgagatttttt
tatctttcatataatggcagagtaaataaataaagtatggcgaaccaataagacaaagatctttcccttgatttttttattcgttcaagactattcaatgttttaacaaaatctattcgtt
cttctgttacggtaaatgtcgtaggatataacaattccaccaaactcataacatatttaaaccaaatattatgagcattaaatctatctggtcccaaattgactaattctatttgatga
tcaaaccaactaccaacatatttcatgccataactcacagagcctaagagtctctcaatacttaatctgtcacgcgccacctgactttttaaaccattcgtgccgctactggtaaacc
aactttcaatctcgttttcctgagaagttaataagcgagtaaacttaaaaaccgatgttgggaatacaggtatgtcatcaatttccgtaatattgtcatctactttgtgtgcctgaca
gtagtgacgatattctcgacaatgtttataatgattacgaaatgcatcaagcacaagtttctttctgatttttttcctgctcgtcgtaagaccacactaatggatcgctcgaaaaaatc
aaatcatcaatttctgagcttgctgtaatttcttgtttatcaacatatgaagtcatacctgttttactcctcaagataatattagaaagtatggcagcactgctgtcatactcttttatac
ccttcatctttcaagctgctgctttgttggctgctttcactcaccccagtcacatagttatctatgctcctggggattcgttcacttgccgcctcgctgcaactcgaaatctattaggta
tattccatgtggtacttcttaatattatcatcaacaatattgattacatttttttggctcatcaaatcattcattggttcaaaggacagcaatacactttcgcaccacacttttcaattgc
caacttagccgcagttatacactccgtataatttccgacagcgttttctgcaattatttcttcaagtttatttcgaaattttcattagggtgcatttcaagaacataatcactaataaat
gcacgcgtctcttgtttagctttattactatcttcgttatagttaactaatatcattaactgatggtctatctctgataggtcaacgtcatatttatccgcaacggctttatatctttcagc
atattcatatctaacatcattagaatcatcccacttaaagatgagaggaatacctttttttggccgcccactcaacaatatgatgactggttgctgttacatatttccgaggtccgcct
ggcgtataagcatggggatttacagatattttagggaagctataaaaatcgttatctggattacaatagcctgttgttaaagcatcgttaatgatttcataacactcttcaaatagt
tgctgttgatattcaaccgggcgattaaaaaaatgcatttcatcttttttttcgcaatcactaaaccctaaaataaatctcccttcacttaactgatccaataagcaagcttcctccgct
atggcgacaggatgatgagttgtaatgatgtgatttaatgaaccaattttaattttctctgttaaaccgagcagaaaaccagaaacagtcagaggagcgccgacaacaccatt
atctgaaaaatgattttcatacactaaaatctgttcaaaattcaacttatcaacatactccgttatttcctgcatgcgaactatactttgttcttgaacagttgttgaattgatgaagtt
aaggaagaacaatccaaatttcatttctttctccttagctaatataatagcgaacgttgtttttctttaagaaatggcatgacatcagactggaagagcttcatggaagcaataatt
tcgtctactgttccattagcttcaaatccacaacaaatatttgatattcctgtagcatcaatgtctttttgaattatgtcaatacattcctgcggcgttcccacgggattgatttcgtaac
tgtaatcaatacggcgattagtatctttatgtccttttaatacaaagtcacgccactgccctttattgaaatcataacctcttgtttggtctgaatcatcaaaaatagtcgtagcattca
cataagaatcataccaatgccccagaaatttccggcaaatctctttcgctttaattgagtcatgatctacagatgttatatatgataagcaatggtcgatattatgaatatcgtgccc
atattcttgagccacttcattataaagctcaagttgtgctttcttttcgttagtatttataatccaacttaatatcatcggtaggccaaattgagcagcccactcagtcgtcgaagctg
attcagccaccacataaaccggtgcgccacctctgctatacgccgcggggtttacttttaccttatggaacttgatatgttcattatcagcttccatatatccctctgtcatgccattct
ttatcagcccgtaccagcattccgctaaggcgcgactgttattcatatctgtgccgaatacgcgaaagtccttgttgtaaagccctcggcaaataccaaaccgaaatcgtcctttt
gacatttgatccaataaattcacatcttcaagttggcgtactggatgggctgtgggaagaacaatagcggcagttcctacattcaatttttttagtcgcgccaagtaaatatgcag
cagcgacataagggttaccaagcaaaccaaactccgtgaaatgatgctccagtaaccatacggtatcaaaaccacactcctcagagatgcgacctaatttaaccaaacgtttc
attacctctgtttgagaaaattggggaggttggtatgtaagcaaaaagtttccaaatttcatagagagtccttatattgctatttgagtgatagaatatctcaatagattttaagac
agagaaattgcttgattttcaatctcaattctcattcggcgttcattgactgtcgcaatagttaaatgttcaaatgacggttcagtaatatcaacatcaatatccagatgatcattatc
catcgcgatagcggctttcgtaaccgattgataaaaaattgcgcaggaccactaaattttcactcaagtcatgcgaacttcctaacaaagaatatatcttgcatcgattactacgaa
tatttgataacaatgtgataacttcatcttgcttgacccaattatcgttatttgcagtaaaagcaataaacggtatatcaagatacacatcatgttattaattgtagaagctaaatcttcc
caaccaaaatcaagacaatctctcgcaaagacttcagcacccaatttatggccttcaaaatctagattatccggcaattcattaatgggtagactgagataatcaaaccctaaag
ctctttcaagagagaatatcttaagttaacaacaccgactgcggtgattaaaaacgaagcattgatttcagataggcttgcataagctatccgcgcagataagcttgaagccaaca
taccgaagttatttatttttcgtgtagttaaccaatcaaccactgctaacaagctctgctttcctatagacattgtaaattcatcaattgtccctgaactcaatccaacgtggtgaagc
gaatcatagcggatcacatgaaatccattccgcgataaatattccgccagaccagcaaaatgatccatcctgcgggcaaaaccagacgcaataataatggcattctttctctttg
ggctgttttcttctggcagcgtttcccaaacatgaatttttttatttccttcaacacaaataacgtggtcgatggtttttatattttgattcattttccatacttttacctattatgggacaaa
tacaaggaacttatcttcttccaggaatcgagtctgttctatttcaaccgcaacatccttagccgtatagttagatggcctttcatgagaaatatatgtcactaatcgttgcaacggt

ctcattccgtcatgagatccaccaactcgaaatatgttattcattcctgcttctacaatcctttccgcaccttttaatgctaacgcatctcgatatttaaatgatgactcccaaggaaaa
atagatatggtttgcgtcttattttttgaacataaggcaatatttgctcaatattatcgacgtgatgaaggtacacacatctgccaagtggttgattaaattccacacctgcatttga
ctcaataatcatccaacgttgatgaatatccacctctacttttaatccagcaaacaagctttctttttgaactaaagaataggccgccttttcatcaaaatctttttggcattcggtaa
tatatgcgcatatagattaagtttttctatcaacgctaacttaaattcctcataatgatttcccatgtaatatatgttttgggcagaaaaacaagctcgctgatcgtaaaaacaaaca
tcatgagccgcacctgtcgctgcggacgtcaaatcaacaggattatcgataatgcaaagactctttttagaaccaaatttaatcacatcagcataagatggcgcatgctctaccg
cccaattaatcgcatctggccctccccaagcgacaataacatccgcatgtcgcataatttcttttgcgagtgatgtatcaccttggtggggccaatatataacagataaagagcg
cgttatcggatgattagggtctacatcaataaaacttaacgctaatgcattagcggtaaaaggatcggttgacgatgtttttataatacactgattcttagttaaaattgcgcgtaa
tatagacatgatcccagataatggaacattacctgccaacagatgtacagatttacctttcggaaaagcccgaacataactttcatcctgaggtagccattcatccatgatatgg
cgagaaccaagttcattttctacaacatcataaaggccgcctttagaacataaaatcatagatatccaattggcctctagcttagccatttcttctgaatatcccatatattttttaag
tcacgaatgtatgtcctgcgtcttgagtattcttcatttttccatctttgccctaccgtatagagaaaattgacaatgttatgcaaccgtaattcgttatttccattacaatcaataatgt
tttttacatgagagtcattcaatattggcaggtaaacactattatcaccaaaattaatggattgcactaaatcatcactttcgggaaagatttcaacctggccgttaataatgaatg
aaatttttttagtcatatttgccatccatttaatgggggtaatttcaacttaagttgctgtttaactatcttgcgaactggtcgtaccaacacttgcttcttatatttttgtgttatgacgat
gtaaataacaaatatatagaaaatcagattctgactacgtggttcctggcctgtaactttcgtcataattatgataataatataaattttctgctcaaggtttttaatctaactgaatg
gatcgtcaatataacgcctattcccgttttaatatctgttaaaatagcaaaggaaaattttcatatcaacaagatttgttaatgaaaagtttaaattaaaaaagtcatgctcttctcta
atgcgtgataatatttatgtaaattattatcctgagtattatttcacaaataccgatagaaatagattgaatagcaatctaatttttaccggcagatttctaaagaagaattcgatatc
aagcttatcgataccgtcgacctcgagggggggcccggtacccagcttttgttccctttagtgagggttaattgcgcgcttggcgtaatcatggtcatagctgtttcctgtgtgaa
attgttatccgctcacaattccacacaacatacgagccggaagcataaagtgtaaagcctggggtgcctaatgagtgagctaactcacattaattgcgttgcgctcactgcccg
ctttccagtcgggaaacctgtcgtgccagctgcattaatgaatcggccaacgcgcggggagaggcggtttgcgtattgggcgcatgcataaaaactgttgtaattcattaagc
attctgccgacatggaagccatcacaaacggcatgatgaacctgaatcgccagcggcatcagcaccttgtcgccttgcgtataatatttgcccatggacgcacaccgtggaaa
cggatgaaggcacgaacccagttgacataagcctgttcggttcgtaaactgtaatgcaagtagcgtatgcgctcacgcaactggtccagaaccttgaccgaacgcagcggtg
gtaacggcgcagtggcggttttcatggcttgttatgactgtttttttgtacagtctatgcctcgggcatccaagcagcaagcgcgttacgccgtgggtcgatgtttgatgttatgg
agcagcaacgatgttacgcagcagcaacgatgttacgcagcagggcagtcgccctaaaacaaagttaggtggctcaagtatgggcatcattcgcacatgtaggctcggccc
tgaccaagtcaaatccatgcgggctgctcttgatcttttcggtcgtgagttcggagacgtagccacctactcccaacatcagccggactccgattacctcgggaacttgctccgt
agtaagacattcatcgcgcttgctgccttcgaccaagaagcggttgttggcgctctcgcggcttacgttctgcccaggtttgagcagccgcgtagtgagatctatatctatgatct
cgcagtctccggcgagcaccggaggcagggcattgccaccgcgctcatcaatctcctcaagcatgaggccaacgcgcttggtgcttatgtgatctacgtgcaagcagattac
ggtgacgatcccgcagtggctctctatacaaagttgggcatacgggaagaagtgatgcactttgatatcgacccaagtaccgccacctaacaattcgttcaagccgagatcgg
cttcccggccgcggagttgttcggtaaattgtcacaacgccgccaggtggcacttttcggggaaatgtgcgcgcccgcgttcctgctggcgctgggcctgtttctggcgctgg
acttcccgctgttccgtcagcagcttttcgcccacggccttgatgatcgcggcggccttggcctgcatatcccgattcaacggccccagggcgtccagaacgggcttcaggcgc
tcccgaaggt
