# Supplementary material for: Application of the Luminescent luxCDABE Gene for the Rapid Screening of Antibacterial Substances Targeting Pseudomonas aeruginosa
Source: Foods. 2023 Jan 13;12(2):392. doi: 10.3390/foods12020392 (PMC9857705; doi:10.3390/foods12020392)
Supplement: Supplementary file 1 [file foods-12-00392-s001.zip › foods-2119381-supplementary/foods-2119381 - Supplementary material S2-done.pdf]

Supplementary Materials S2

Application of the Luminescent *lux*CDABE Gene for the Rapid Screening of Antibacterial Substances Targeting *Pseudomonas aeruginosa*

Yue Peng [1,†], Qian Wang [1,2,†], Kaixiang Zhu [3] and Wu Ding [1,*]

[1] College of Food Science and Engineering, Northwest A&F University, Yangling 712100, China
[2] Academy of National Food and Strategic Reserves Administration, Beijing 100037, China
[3] College of Life Sciences, Northwest A&F University, Yangling 712100, China
* Correspondence: ding_wu@nwsuaf.edu.cn; Tel.: +86-15002926160
† These authors contributed equally to this work.

Detailed transformations procedures

STEP 1: Extraction and validation of pBBR1MCS-4-luxCDABE

The extraction of pBBR1MCS-4-*lux*CDABE was completed using an OMEGA E.Z.N.A.® Plasmid Mini Kit I, (V-spin) kit (Georgia, USA), and the specific steps are in accordance with the instructions. The enzyme digestion validation system performs single and double enzyme digestion in 37°C metal bath for 30 min according to the ratio in Table S2-1. Enzyme digestion product (6 μL) mixed with loading buffer (1μL) was applied to 1.5% agarose gel for 45 min under 130 kV voltage, and then observed under the ultraviolet transmission of gel imaging system.

Table S1. Single and double enzyme digestion system (μL).

Ingredient	Single enzyme digestion system	Double enzyme digestion system
*Bam*H I	0	1
Xho I	1	1
10×NE Buffer	2	2
DNA	3	3
ddH_2O	To 20 μL	To 20 μL

STEP 2: Construction of pBBR1MCS-5-luxCDABE

The products of pBBR1MCS-5 and pBBR1MCS-4-*lux*CDABE after double enzyme digestion according to the ratio in Table S2-2 were used for this operation. Purification and recovery of enzyme digestion products was completed using an OMEGA E.Z.N.A.® Gel Extraction Kit (V-spin) kit (Georgia, USA), and the specific steps are in accordance with the instructions. A NanoDrop™ One micro ultraviolet visible spectrophotometer (Thermo Scientific, USA) was used to measure the nucleic acid concentration to determine the addition ratio of each segment in the enzyme-linked system. The purified product is connected by T4 ligase, and the connection is completed by 22°C metal bath for 2h as Table S2-2.

Table S2. DNA linkage system (μL).

Ingredient	Volume
Digestion of pBBR1MCS-5 fragment	100 ng
Digestion of *lux*CDABE fragment	5 times volume of the plasmid
10×T4 DNA ligase Buffer	2
50% PEG 4000 solution	2
T4 DNA ligase	1

ddH_2O	To 20 μL

STEP 3: Preparation of E.coli TG1 competent cells and transformation of $CaCl_2$

The prepared TG1 seed culture medium was transferred to 50 mL LB (150 mL Erlenmeyer flask) at 1:100. It was cultured to the initial stage of the logarithmic phase after about 2~3 h (37°C, 180 rpm). At this time, the cell shakes like a floating cloud (Note: The culture time should not be too long, or the cells will become turbid and cannot be used). 1.5 mL of bacterial solution was centrifuged at room temperature and the supernatant was discarded (4000 rpm, 5 min). Repeat the operation once. The mix solution was centrifuged at 4°C and the supernatant was discarded (4000 rpm, 5 min). 150 μL $CaCl_2$ (0.2 M, pre cooled) was added into the system, blown and mixed evenly, then placed in ice bath for 2 h. Finally, 50 μL glycerine (50% v/v) was added into the system and then mixed evenly. Each 1.5 mL sterile centrifuge tube is filled with 100 μL competent cells. Excess TG1 competent cells can be frozen at - 80°C for use at the next time.

Add all the enzyme linked products (STEP 2) into a tube of TG1 competent cells (100 μL), gently blow and mix, and take ice bath for 30 min. the heat shock was completed by ice bath for 1-5 min after heat shock (42°C, 90 s). The above products are transferred to 900 μL SOC medium (preheated to 37℃) for 1 h (220 rpm, 37℃). The mix solution was centrifuged at room temperature and the 800μL supernatant was discarded (4000 rpm, 5 min). The residuals resuspended and coated on LB solid plate (Km: 50 μg/mL). After incubation at 37°C for 10 h, the plate was placed in the Bio-Rad imaging system and photographed in the full dark room to verify that it could emit light. Several single colonies with high luminescence intensity were selected for subculture, and the recombinant luminescent *E. coli* with the highest brightness was screened by imaging system and named TG1-CE. A characteristic sequence of *luxD* on *lux*CDABE was used as template to design primers (Table S2-3), and bacterial solution PCR was performed on TG1-CE to verify whether pBBR1MCS-5-*lux*CDABE was successfully introduced into the target strain. The PCR system was listed in Table S2-4. The circulation steps are: 95°C, 5 min; 95°C, 30 s, 50°C, 30 s, 72°C, 90 s, 32 cycles; 72°C, 10 min.

Table S3. Primer sequence of *LuxD.*

Target gene fragment	Primer	Primer sequence(5′→3′)
Characteristic fragment of *LuxD*, about 1600bp	*LuxD*-F	ATGGAAAATGAATCAAAATATAAAACC
	LuxD-R	TCAAGTTGTGCTTTCTTTTCGTTAG

Table S4. *LuxD* PCR cycle system of TG1-CE.

Ingredient	Volume
2×Es Taq MasterMix (Dye)	10
*Lux*D-F	0.5
*Lux*D-R	0.5
Template bacterial solution	2
ddH_2O	To 20 μL

STEP 4: Extraction and validation of pBBR1MCS-5-luxCDABE

Detailed operation were same as STEP 1.

STEP 5: Preparation and electrotransformation of competent cells of P. aeruginosa

The prepared PAO1 seed culture medium was transferred to 50 mL LB (150 mL Erlenmeyer flask) at 1:100. It was cultured to the initial stage of the logarithmic phase after about 3~3.5 h (37°C, 180 rpm). At this time, the cell was just not clear from the side view (Note: The culture time should not be too long, or the cells will become turbid and cannot be used). The bacterial suspension was placed in ice bath for 30 min, and then sub packed into sterile 50 mL centrifuge tubes, and washed twice with pre cooled 300 mM sucrose

solution of equal volume (4°C, 4000 rpm, 10 min). Resuspend the cell with the residual sucrose solution in the tube wall. 100 μL bacterial suspension was transferred to 1.5 mL EP tube, placed on ice to cool for 15 min, then the competent cells of *P. aeruginosa* was obtained and must use immediately.

10 μL pBBR1MCS-5-*lux*CDABE (STEP 4) was added into competent cells of *P. aeruginosa*, gently and rapidly blew and mixed, and then transferred to an pre cooled electric rotating cup. The mixed solution was shocked under the voltage of 1.9 kV, and the system after electroporation was transferred to 5 mL LB for resuscitation and culture for 3 h (37°C, 180 rpm). Accurately transfer 1.5 mL of bacterial solution into 1.5 mL EP tube, and centrifuge at 4000 rpm at room temperature for 3 min. Discard the supernatant, and suspend and coat the rest on LB solid plate (Km: 50 μg/mL，Gm: 100 μg/mL）. The coated plate was placed in a 37℃ constant temperature incubator for 36 h, and then photographed with full dark condition in the Bio-Rad imaging system.

STEP 5: Validation and screening of recombinant P. aeruginosa

The single colony of several recombinant luminescent *P. aeruginosa* with high luminescence intensity was selected respectively to culture in LB, and the successful construction of recombinant luminescent *P. aeruginosa* was verified by Bio rad imaging system and bacterial solution PCR (Table S2-5).

Table S5. *Lux*D PCR cycle system of PAO1-CE and PA27853-CE (μL).

Ingredient	Volume
2×Es Taq MasterMix (Dye)	10
*Lux*D-F	0.5
*Lux*D-R	0.5
Template bacterial solution	1
ddH_2O	To 20 μL
